# Drug-Loaded Silver Nanoparticles—A Tool for Delivery of a Mebeverine Precursor in Inflammatory Bowel Diseases Treatment

**DOI:** 10.3390/biomedicines11061593

**Published:** 2023-05-30

**Authors:** Mina Todorova, Miglena Milusheva, Lidia Kaynarova, Deyana Georgieva, Vassil Delchev, Stanislava Simeonova, Bissera Pilicheva, Stoyanka Nikolova

**Affiliations:** 1 Department of Organic Chemistry, Faculty of Chemistry, University of Plovdiv, 4000 Plovdiv, Bulgaria; minatodorova@uni-plovdiv.bg (M.T.); or miglena.milusheva@mu-plovdiv.bg (M.M.); 2 Department of Bioorganic Chemistry, Faculty of Pharmacy, Medical University of Plovdiv, 4002 Plovdiv, Bulgaria; 3 Department of Analytical Chemistry and Computer Chemistry, Faculty of Chemistry, University of Plovdiv, 4000 Plovdiv, Bulgaria; l.kainarova@uni-plovdiv.bg (L.K.); georgieva@uni-plovdiv.bg (D.G.); 4 Department of Physical Chemistry, Faculty of Chemistry, University of Plovdiv, 4000 Plovdiv, Bulgaria; 5 Department of Pharmaceutical Sciences, Faculty of Pharmacy, Medical University of Plovdiv, 4002 Plovdiv, Bulgaria; 6 Research Institute, Medical University of Plovdiv, 4002 Plovdiv, Bulgaria

**Keywords:** silver nanoparticles, mebeverine precursor, green method, galactose, inflammatory bowel diseases

## Abstract

Chronic, multifactorial illnesses of the gastrointestinal tract include inflammatory bowel diseases. One of the greatest methods for regulated medicine administration in a particular region of inflammation is the nanoparticle system. Silver nanoparticles (Ag NPs) have been utilized as drug delivery systems in the pharmaceutical industry. The goal of the current study is to synthesize drug-loaded Ag NPs using a previously described 3-methyl-1-phenylbutan-2-amine, as a mebeverine precursor (MP). Methods: A green, galactose-assisted method for the rapid synthesis and stabilization of Ag NPs as a drug-delivery system is presented. Galactose was used as a reducing and capping agent forming a thin layer encasing the nanoparticles. Results: The structure, size distribution, zeta potential, surface charge, and the role of the capping agent of drug-loaded Ag NPs were discussed. The drug release of the MP-loaded Ag NPs was also investigated. The Ag NPs indicated a very good drug release between 80 and 85%. Based on the preliminary results, Ag NPs might be a promising medication delivery system for MP and a useful treatment option for inflammatory bowel disease. Therefore, future research into the potential medical applications of the produced Ag NPs is necessary.

## 1. Introduction

Inflammatory bowel diseases (IBDs) are functional chronic multifactorial diseases of the gastrointestinal tract with a complex pathogenesis and multifaceted therapy approaches, aimed at alleviating clinical symptoms and improving the life quality of patients. IBDs are becoming more widespread globally, although the actual cause is still unknown. Their treatment includes dietary changes and uptake of drugs from various pharmacological groups such as antidiarrheals, prokinetic agents, bulk-forming laxatives, tricyclic antidepressants, anticholinergics, serotonin receptor antagonists, and newer pharmacological classes targeting chloride ion channels and guanylate cyclase C [1]. Chronic inflammation most often affects the colon or small and large intestines [2]. Despite the fact that some of the currently available treatments for ulcerative colitis are helpful, there are still certain restrictions on their use due to their nonspecific distribution, gastrointestinal drug metabolism, and considerable side effects [3]. A well-designed drug delivery system is beneficial to enhance therapeutic efficacy, such as the use of drug-release system, prodrugs, system affected by pH, micro- and nanoparticulate systems, etc. [4,5]. A number of medications, including corticosteroids, antibiotics, and immunosuppressants, have recently been shown to support mucosal healing. Innovative platforms based on biomaterials with greater efficacy and fewer adverse effects are required to deliver pharmaceutical medications to the wounded location. They can be used as effective drug delivery systems to facilitate colon-specific administration and stable drug release [6]. Drug delivery methods to the micro- or nanometer scale might prolong colonic residency duration but also have more advantages for treating IBD. A size-dependent accumulation of micro- and nanoparticles, especially in the inflamed intestinal regions, can be observed [7,8]. That makes them one of the best options for drug delivery regulation.

The synthesis of nanomaterials is an essential aspect of nanotechnology. Nanoparticles (NPs) of noble metals have been known for the last decades [9] due to their expanding number of pharmaceutical and biomedical applications, such as drug delivery, photothermal therapy, radiotherapy, imaging, etc. [10,11]. Amongst the different noble metal NPs, silver and gold NPs are mostly synthesized so far due to their remarkable biological activities and unique physicochemical properties [12,13,14,15,16,17,18,19,20,21,22,23,24,25,26,27]. Typically, they are synthesized using hazardous chemicals which affect the environment and human health. This necessitated the development of environment-friendly methods and gave rise to the green synthesis methodology [28,29].

Critical objectives in modern synthetic organic chemistry include the improvement of reaction efficiency, the avoidance of toxic reagents, the reduction of waste, and the responsible utilization of our resources. Starting from this assumption, the search for a green alternative to produce NPs or the discovery of green molecules is a challenge in order to obtain safe materials [30]. The biological techniques include the use of different biological resources as reducing and stabilizing agents to change the noble metal cations to their nanoforms [31,32,33,34].

Recently, the application of carbohydrates has become a popular area in NPs synthesis. Many interesting methods are being applied currently to the green preparation of nanosized silver nanoparticles (Ag NPs) such as the biosynthesis of nanoparticles by starch [35,36,37], by plant leaf broth [38], by edible mushroom extract [39], etc. Green syntheses of Ag NPs with polyoxometallates, polysaccharides, Tollens, Fehling’s, irradiation, etc., were reported as well [40,41]. Polymer-based NPs were synthesized due to the spectrum of biological interest [42]. Sugars as reducing agents for the green synthesis of metal NPs were applied in the last ten years [43,44,45,46,47]. Many mono-, di-, and polysaccharides were used as reducing and capping agents for the preparation of noble metals NPs due to their sustainability, abundance, low cost, harmlessness, renewability, biodegradability, and compatibility with biological systems [48,49,50,51,52]. To prevent aggregation, the capping agents were applied. The capping agents are most often organic molecules that bind the metallic core, developing a layer on the surface of NPs. Many capping agents have been used in conventional syntheses, such as cetyltrimethylammonium bromide, polyvinylpyrrolidone, oleic acid, sodium dodecyl sulfate, tetraethyl ammonium bromide, etc. [53,54,55]. These reagents have an effective role in NPs growth and control of their size but could be hazardous [56], causing many doubts about their use in biological applications [57]. The reducing agents commonly used in a chemical route are hydrazine, formaldehyde, and sodium tetrahydridoborate [58], which are toxic to the environment and living organisms. Other typical capping and reducing agents are sodium citrate and tannic acid, which are used to obtain highly monodispersed NPs [30,59].

The major challenge of using Ag NPs as drug-delivery systems, particularly in the treatment of inflammatory bowel disease, is carrying out site-specific drug delivery to the colon. Contrarily, long-term medicine has numerous adverse effects that are detrimental to the patients’ quality of life, causing diarrhea, ulcers, or vomiting [60,61,62,63]. Targeting the medications directly to the colonic region of the gastrointestinal tract is the main problem facing researchers [64]. Another obstacle to overcome is the retardation of skeletal and growth development brought on by an inadequate diet. Cytokines and other inflammatory mediators lead to changes in the hormonal axis that have an immediate impact on growth. A proper diet and the correct anti-inflammatory treatment can help with these issues.

**Figure 1:**
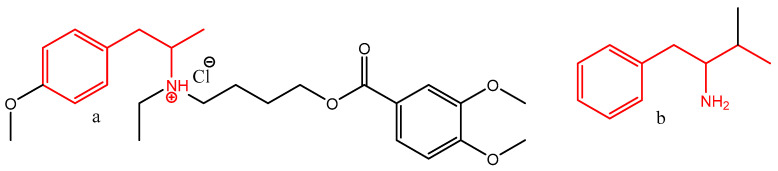
Structure of mebeverine hydrochloride (**a**) and the mebeverine precursor (MP) (**b**) (MP common structure in red).

The quest for NPs synthesis and their application as drug-delivery systems prompted us to obtain Ag NPs as drug-delivery systems for recently described antispasmodics in new therapeutic approaches for irritable bowel syndrome treatment [65]. Recently, we explored the synthesis of a mebeverine precursor (MP, 3-methyl-1-phenylbutan-2-amine, Figure 1b) based on its pharmacological properties. Our decision was also driven by the structural similarity to mebeverine (Figure 1a), a second-generation papaverine analogue with a history of pharmacological applications as an IBD treatment.

**Scheme 1:**
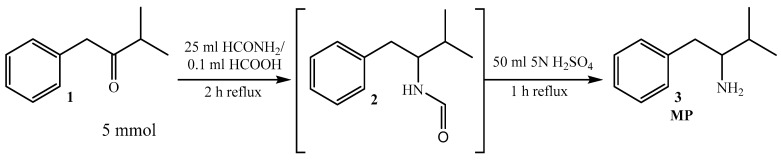
Reaction pathway for the synthesis of MP.

In order to explore their biological activity, we synthesized many MPs with a C-3 substituent [65] (Scheme 1).

In the present study, we focused our investigation on the synthesis of drug-loaded Ag NPs with the most active MP (**3**, Figure 1b). The MP structure was chosen as the leader compound with favorable antimicrobial, immunohistochemical, and spasmolytic activity, and a reasonable effective choice for orally active long-term therapy of chronic IBD [65].

## 2. Materials and Methods

All solvents and reagents were purchased from Merck (Merck Bulgaria EAD, Darmstadt, Germany). Melting point was determined on a Boetius hot stage apparatus and was uncorrected. IR spectra were determined on a VERTEX 70 FT-IR spectrometer (Bruker Optics, Ettlingen, Germany). MP was characterized by ^1^H NMR, ^13^CNMR, IR, and HRESIMS. The purity was determined by TLC using several solvent systems of different polarity. TLC was carried out on precoated 0.2 mm Fluka silica gel 60 plates (Merck Bulgaria EAD), using chloroform: diethyl ether: n-hexane = 6:3:1 as a chromatographic system. Neutral Al_2_O_3_ was used for column chromatographic separation. The product, after evaporation of the solvent, was purified by recrystallization from diethyl ether.

### 2.1. Synthetic Protocol

#### 2.1.1. Synthesis of MP [65]

To a solution of 5 mmol of the starting ketone 3-methyl-1-phenylbutan-2-one **1** in 25 mL formamide, a catalytic amount of methanoic acid was added. The mixture was refluxed for 2 h at 180 °C, then poured into water and extracted with CH_2_Cl_2_ (3 × 20 mL). The combined extracts were washed with Na_2_CO_3_ solution, water, and dried using anhydrous Na_2_SO_4_, filtered on the short column filled with neutral Al_2_O_3_, and then concentrated. The obtained formamide was directly hydrolyzed with 50 mL 5N H_2_SO_4_ and 1 h reflux at 100 °C to 3-methyl-1-phenylbutan-2-amine (**3**, MP). The mixture then was poured into water and extracted with CH_2_Cl_2_ (2 × 20 mL). The water layer was alkalized with NH_4_OH and extracted with CH_2_Cl_2_ (3 × 20 mL). The combined extracts were dried using anhydrous Na_2_SO_4_, filtered on the short column filled with basic Al_2_O_3,_ and then concentrated. Spectral data confirmed the structure of the MP.

3-methyl-1-phenylbutan-2-amine (MP) ^1^H-NMR: 0.98 (dd, J = 9.8, 6.8, 6H, 2×CH_3_), 1.35 (broad s, 2H, NH_2_), 1.67 (dq, J = 10.8, 6.8, 1H, CH(CH_3_)_2_), 2.40–2.43 (m, 1H, CHNH_2_), 2.81–2.86 (m,2H, CH_2_), 7.19–7.22 (m, 3H, Ar), 7.28–7.31 (m, 2H, Ar); ^13^C-NMR: 140.3 (Ar), 129.2 (Ar), 128.5 (Ar), 126.1 (Ar), 58.2 (CHNH_2_), 41.3 (CH_2_), 33.1 (CH(CH_3_)_2_), 19.4 (CH(CH_3_)_2_), 17.5 (CH(CH_3_)_2_). IR(KBr) ν_max_, cm^−1^: 3419 ν(N–H), 3071, 3061, 3030 ν(C-H, Ph), 2960 ν^as^(C–H, CH_3_), 2938 ν^as^(C–H, CH_2_), 2898 ν^s^(C–H, CH_3_), 1627 δ(NH_2_), 1590, 1571 ν(C=C, Ph), 1494 δ(CH_2_), 1464 δ^as^(CH_3_), 1375 δ(CH_3_, i-Pr); HRMS (ESI) *m*/*z* 164.14323.

#### 2.1.2. Synthesis of Galactose-Assisted Ag NPs

A total of 1.25 g (0.007 mol) galactose was dissolved in 25 mL water and refluxed for 5 min; then 0.63 mL of 0.01 M AgNO_3_ solution was added, and the mixture was kept at boiling. In about 5 min, the color of the solution turned pale yellow, indicating the formation of Ag NPs.

#### 2.1.3. Synthesis of Galactose-Assisted MP-Loaded Ag NPs

A total of 1.25 g (0.007 mol) galactose was dissolved in 25 mL water and refluxed for 2 min; then 0.63 mL of 0.01 M AgNO_3_ solution was added. To find out how the ratio of Ag NPs to the drug molecule would affect the drug release, different amounts of MP were used in the preparation of the solutions. Throughout the trials, Ag NP concentration was constant, whereas drug concentration changed depending on the ratio. The chosen ratios of drug molecules to Ag NPs were 1:1, 1:3, and 1:6, respectively. The solution’s color changed to a light yellow after roughly 5 min of reflux, signifying the generation of Ag NPs.

### 2.2. Characterization of the Ag NPs. Analytical Techniques

After NPs preparation, the solution was used for UV-Vis, transmission electron microscopy (TEM), single-particle ICP-MS (sp-ICP-MS), dynamic light scattering (DLS), and zeta potential. For Fourier transform infrared spectra (FTIR) and X-ray diffraction (XRD) analysis, the suspension was centrifuged at 5000 rpm for 15 min and filtered (0.22 µm, Chromafil^®^, Macherey-Nagel, Düren, Germany), and the precipitate was used.

#### 2.2.1. UV-Vis Spectra

UV–Vis spectra were recorded in the range between 320 and 800 nm using a Cary-60 UV-Vis spectrophotometer (Agilent Technologies, Santa Clara, CA, USA). Solution spectra were obtained by measuring the absorption of the prepared nanoparticle dispersions in a quartz cuvette with a 1 cm optical path.

#### 2.2.2. FTIR Spectra

IR spectra were determined on a VERTEX 70 FT-IR spectrometer (Bruker Optics, Ettlingen, Germany). The spectra were collected in the range from 600 cm^−1^ to 4000 cm^−1^ with a resolution of 4 nm and 20 scans. The instrument was equipped with a diamond attenuated total reflection (ATR) accessory. The IR spectra were analyzed with the OPUS-Spectroscopy Software, Bruker (Version 7.0, Bruker, Ettlingen, Germany).

#### 2.2.3. spICP-MS

A 7700 Agilent ICP-MS spectrometer (Agilent Technologies, Tokyo, Japan) equipped with MicroMist™ nebulizer and Peltier cooled double-pass spray chamber was used for the characterization of silver nanoparticles at 107 amu. The ICP-MS operating parameters were as follows: RF power—1.55 kW; sample flow rate—0.336 mL min^−1^; carrier Ar gas flow rate—1.2 L min^−1^; acquisition time 60 s; dwell time 5 ms; and transport efficiency 0.032. The transport efficiency was determined by the particle-size method [66]. Ultrapure water (UPW) was used throughout the experiments (PURELAB Chorus 2+ (ELGA Veolia) water purification system). For the sonication of silver colloids, an ultrasonic bath (Kerry US) was used. Reference materials (RMs) of citrate-stabilized silver dispersions Ag NPs (Merck Bulgaria EAD) with mean size 40 ± 4 nm and total mass concentration of silver 0.02 mg mL^−1^ were used in this study for transport efficiency determination and calibration.

A standard solution of Ag 9.974 ± 0.041 mg L^−1^ in 2% HNO_3_ (CPAchem Ltd., Bogomilovo, Bulgaria) was used for the preparation of ionic standards.

#### 2.2.4. TEM

The morphology, shape, and size of the nanoparticles were investigated by TEM (Talos 1.15.3, Thermo Fisher Scientific, Waltham, MA, USA). The nanoparticles suspension was added dropwise onto a formvar/carbon-coated copper grid, and then the TEM observation of the samples was performed at an operating voltage of 200 kV.

#### 2.2.5. DLS and Zeta Potential

DLS measurements were performed on a Brookhaven BI-200 goniometer with vertically polarized incident light at a wavelength l = 632.8 nm supplied by a He–Ne laser operating at 35 mW and equipped with a Brookhaven BI-9000 AT digital autocorrelator. The scattered light was measured for dilute aqueous dispersions in the concentration range 0.056–0.963 mg mL^−1^ at 25, 37, and 65 °C. Measurements were made at θ angles in the range of 50–130°. The system allows measurements of ζ-potential in the range from −200 mV to +200 mV. All analyses were performed in triplicate at 25 °C.

#### 2.2.6. X-ray Diffraction (XRD)

The degree of crystallinity of the synthesized nanoparticles was studied by X-ray powder diffractometry. The diffraction patterns of Ag NPs (blank) and drug-loaded Ag NPs were recorded at a 2θ range from 10° to 80° using a SIEMENS D500 X-ray powder diffractometer (KS Analytical Systems, Aubrey, TX, USA). All the measurements were performed at a voltage of 35 kV and a current of 25 mA. The monochromatic X-rays (1.5406 Å) were generated by Cu-anticathode (K_α1_).

### 2.3. In Vitro Drug Release

In vitro release of MP from the Ag NPs was performed using the dialysis bag method. A dialysis membrane (MWCO 12 kDa, Sigma-Aldrich, St. Louis, MO, USA) was hydrated in distilled water for 24 h. An accurately weighed amount of drug-loaded NPs (equivalent to the amount of MP in ratios of 1:1, 1:3, and 1:6) was dispersed in 10 mL of phosphate buffered saline (PBS) and then transferred to the dialysis bag, which was closed using a plastic clamp. Each bag was placed into a beaker containing 40 mL PBS (pH 7.4, dialysis medium). Aliquots (2 mL) from the dialysis medium were taken for measurements and subsequently replaced at predetermined time intervals with fresh medium. The drug-release study was performed for 24 h. The mean results of triplicate measurements and standard deviations were reported. The solution in the Falcon tube was shaken before each evaluation of UV–visible absorbance. For data analysis, the MP’s maximum absorption band values (λ = 192 nm) were employed. Additionally, drug-only controls (one drug control for each ratio) were made.

## 3. Results and Discussion

The accumulation of nanoparticles in the colon region is one of the most important features of an effective nanomedicine for colon diseases. In the present study, galactose-assisted drug-loaded Ag NPs were synthesized using a direct rapid, entirely green one-pot method. Ag NPs were chosen due to a variety of biological activities [67,68,69,70,71,72,73,74]. In addition, more important is their protective effect on gastrointestinal tract [75]. Galactose was used as a reducing and capping agent. It can quickly reduce Ag^+^ to the ground state at the boiling point of water (100 °C) [45]. Galactose-capped Ag NPs were chosen for the lower toxicity [76,77], so they can be recognized as more biocompatible and therefore are more suitable for exploitation in medical applications. With favorable spasmolytic, antibacterial, immunohistochemical activity and lack of cytotoxicity, the MP structure was selected as a viable and effective option for orally active long-term therapy of chronic IBD [65].

### 3.1. Characteristics of Galactose-Assisted Ag NPs Compared to Galactose-Assisted Drug-Loaded Ag NPs

**Figure 2:**
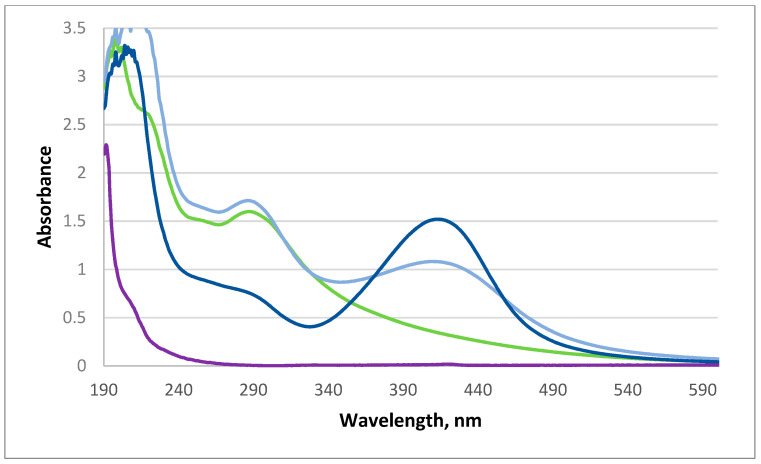
Absorption spectra of galactose (green), MP (purple), Ag NPs (blue), and drug-loaded Ag NPs (dark blue).

Initially, the synthesis of the Ag NPs in an aqueous solution was monitored by recording the absorption spectra at a wavelength range of 190–600 nm (Figure 2). The absorption maximum in galactose solution was detected at 287 nm and MP was observed a 192 nm. In the early 10 min of the Ag NPs formation process, the UV–Vis spectrum included the appearance of a single, strong, and broad surface plasmon absorbance at 412 nm. The surface plasmon resonance effect causes an absorbance band in the electromagnetic spectrum, usually in the visible light range. Particle size and shape, coating, particle spacing, and other variables can all have an impact on the peak [78,79,80,81]. The fact that we observed the peak at the region 410–450 nm corresponds with the literature data for spherical nanoparticles [45,82,83]. The absorption spectra obtained from all the prepared solutions after 10 min are shown in Figure 2.

A hypochromic effect of the absorption maximum of galactose at 287 nm was observed for MP-loaded Ag NPs. In the meanwhile, a hyperchromic effect of the absorption maximum at 412 nm was also seen. The observed effects were proportional to the increased concentration of Ag NPs in the presence of the MP [84].

The UV–visible spectra showed an application of a simple one-pot galactose-assisted green method for the synthesis and stabilization of Ag NPs as drug-delivery systems. The symmetrical plasmon band of MP-loaded Ag NPs indicated the low degree of aggregation [85]. We presume that the lack of aggregation is due to the presence of an isopropyl group in the structure of the MP. Since the intensity of an absorption peak is proportional to the concentration of Ag NPs in the colloidal solution, the appearance of such well-defined peaks suggests that the production yield of our method was quite good.

**Figure 3:**
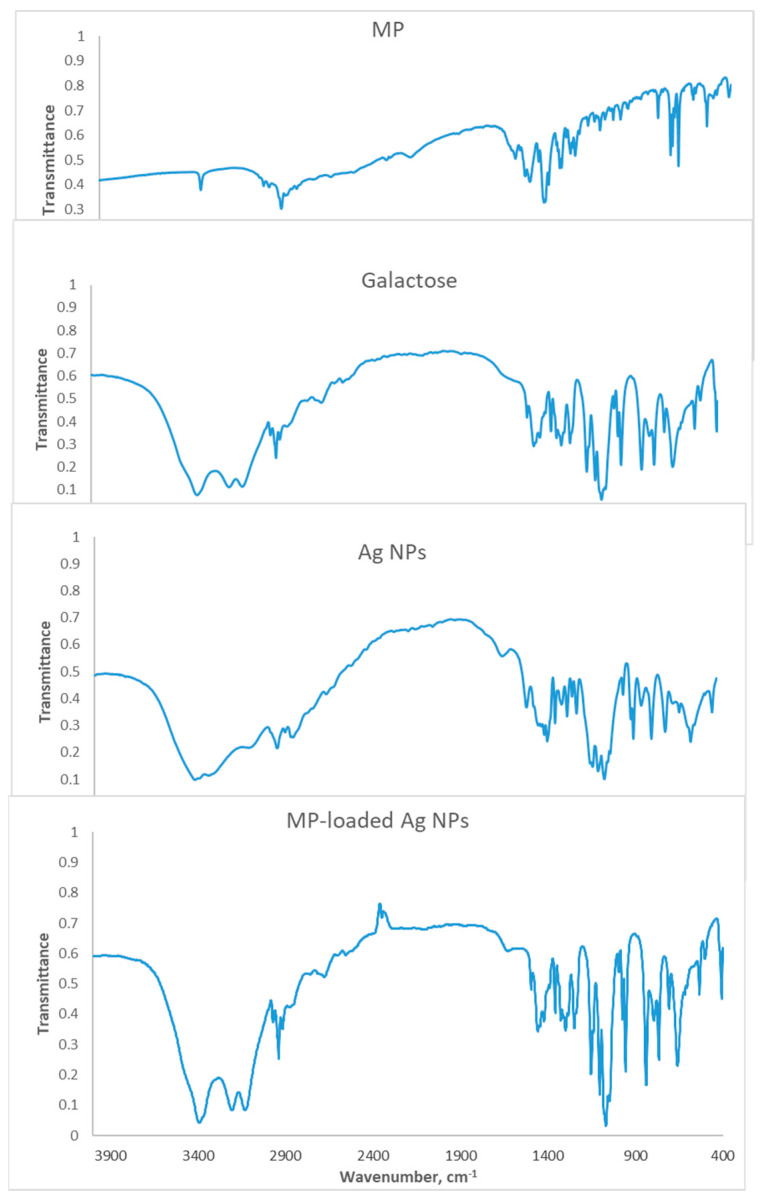
IR spectra of MP, galactose, galactose-assisted Ag NPs, and MP-loaded Ag NPs in the region 4000–400 cm^−1^.

The stability of drug-loaded Ag NPs was also investigated by FTIR and compared to MP, galactose, and galactose-assisted Ag NPs spectrum (Figure 3). IR analysis depicted shifts in the characteristic peaks of galactose, Ag NPs, and MP-loaded Ag NPs, which indicates the interactions between the molecules. The shifts were observed for stretching vibration ν(O–H) from 3386, 3205, and 3131 cm^−1^ in galactose compared to 3421 cm^−1^ for Ag NPs and 3382, 3203, and 3124 cm^−1^ for MP-loaded Ag NPs. Shifts could be found also for the stretching C–H vibration ν(C–H) at 2949, 2937, and 2916 cm^−1^ for galactose compared to 2939–2849 cm^−1^ region Ag NPs and 2972 cm^−1^ for drug-loaded Ag NPs. The deformation vibrations (scissoring δ, wagging ω, and torsion τ) of the CH_2_ group were also shifted from 1457–1248 cm^−1^ in IR of galactose to 1500–1238 cm^−1^ for the IR of Ag NPs, and 1456–1245 cm^−1^ for MP-loaded Ag NPs. The stretching vibrations ν(C–O), ν(C–C), and in-plane bending β(COH) in the pyranose ring of galactose were observed at 1151, 1104, 1067, and 1045 cm^−1^ [86], while the Ag NPs showed peaks at 1119 and 1049 cm^−1^ and MP-loaded Ag NPs—at 1103 cm^−1^. The IR spectrum of galactose and MP-loaded Ag NPs showing the band at 837 cm^−1^ indicates the presence of the α-anomer, while for galactose-assisted Ag NPs, two bands appeared at 836 cm^−1^ and 883 cm^−1^, corresponding to both the anomers—α and β [87,88]. The shifts of the bands in the IR spectra of galactose-assisted Ag NPs and MP-loaded Ag NPs relative to the bands in the IR spectrum of galactose are most indicative of the adsorption and modification of Ag NPs on the surface of the carbohydrate [89].

The depicted shifts in the IR spectra of galactose and MP compared to Ag NPs and MP-loaded Ag NPs confirmed that galactose molecules participate with their C–O and glycosidic OH groups in the formation and stabilization of the Ag NPs [90].

Carbohydrate-assisted (by reduction of glucose, galactose, maltose, and lactose) synthesis of silver NPs in the presence of [Ag(NH_3_)_2_]^+^ obtained Ag NPs with controllable sizes [91]. It is well known that, irrespective of the approach, biomolecules are involved in the reduction of silver nanoparticles, and also have an interaction with the upper face of silver, which is their initial connection of originating particles [92,93]. The coating of silver nanoparticles decreases the agglomeration rate and size [94]. The dynamic surface site of the silver nanoparticles persisted through the particle size, shape, and accumulation rate. The reaction conditions such as temperature, pH, extract volume, reactant concentration, and time describe the size and gradation of the growing particles and, thus, influence the shape and size of the silver accumulates [95]. Silver nanoparticles less than 10 nm may elapse by the nuclear opening and interact with genetic material. These crystals are appropriate for diagnostics and gene therapy, but they are genotoxic. The shape of the nanoparticles is shown to affect cytotoxicity, e.g., plate-shaped silver nanoparticles have revealed higher toxicity as compared to wires or spherical nanoparticles [96,97,98,99].

To establish the size and shape of the Ag NPs, spICP-MS, DLS, and zeta potential were used.

The nanocolloid suspensions were examined by spICP-MS to determine the size and distribution by size of the silver core in synthesized galactose-assisted Ag NPs and MP-loaded Ag NPs. The information about Ag core size is obtained by single-point calibration with RM of citrate-stabilized Ag NPs with size 40 ± 4 nm and one ionic standard of Ag^+^ (1 ng L^−1^) for sensitivity determination.

The introduction of a sufficient number of particles and a 10% likelihood of particle coincidence at the chosen dwell duration were ensured by gravimetrically diluting nanocolloid suspensions with UPW to DF = 5 × 105. Before each dilution step and during the instrumental measurements, the NPs were subjected to an ultrasound treatment for 10 min in order to achieve a uniform dispersion in the solutions.

**Figure 4:**
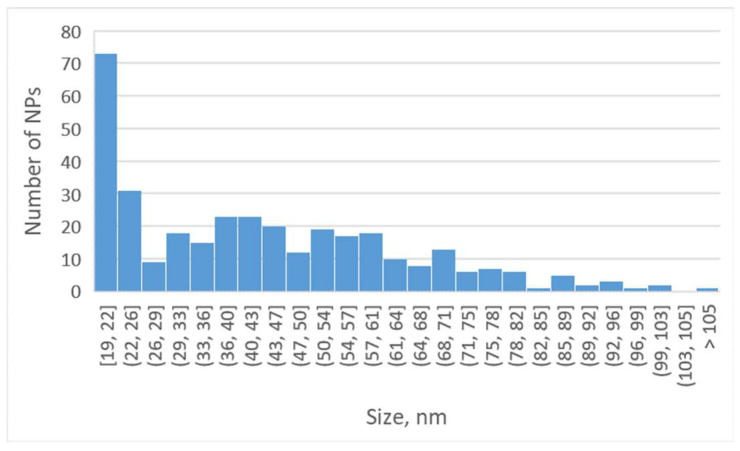
Particle size distribution histogram of galactose-assisted Ag NPs (~400 NPs).

The histogram size distribution for galactose-assisted Ag NPs, presented in Figure 4, shows that the particle size distribution is asymmetric with a high fraction of small particles (19–22 nm), which is close to the method’s detection limit—LODsize 18 nm. The estimated mean diameter for this sample is 42 ± 1 nm, while the most frequent diameter is 20 nm. The results received for MP-loaded Ag NPs show that most detected particles have a size near or below the determined method detection limit (LODsize 18 nm).

**Figure 5:**
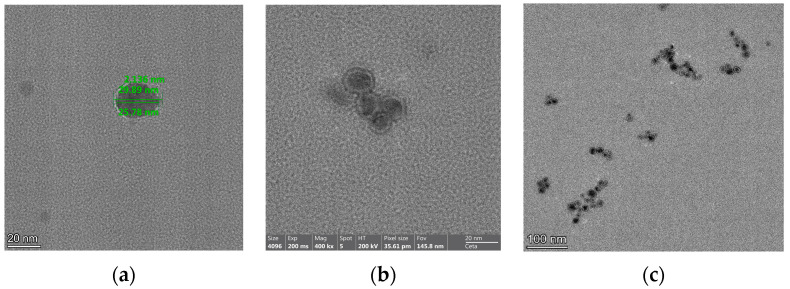
TEM images of galactose-assisted Ag NPs (**a**,**b**); MP-loaded Ag NPs (**c**).

The results from the size distribution histograms (Figure 4) were confirmed by the TEM images (Figure 5a,b). The obtained results clearly indicate the shape and size of the nanoparticles and illustrate the individual nanoparticles as well. The TEM image confirms the synthesis of smaller spherical particles with different size between 10 and 26 nm for galactose-assisted Ag NPs and between 10 and 17 nm size for the drug-loaded Ag NPs. This explains the fact that we cannot quantify the MP-loaded Ag NPs using the spICP-MS.

The TEM images show that the galactose participated as a capping and protecting agent. A thin, galactose-based coat with an average size of 2.136 nm can be observed in TEM images (Figure 5b,c). Many groups such as hydroxyl, carboxyl, phenol, and carbonyl are linked with oxygen and nitrogen with covalent bond linkage for the complex formation of silver, and so they are probably absorbed on its surface [94]. We assume that galactose in galactose-assisted Ag NPs (Figure 5a,b), as well as the isopropyl group of MP in MP-loaded Ag NPs (Figure 5c,d), prevent the aggregation of the particles.

**Figure 6:**
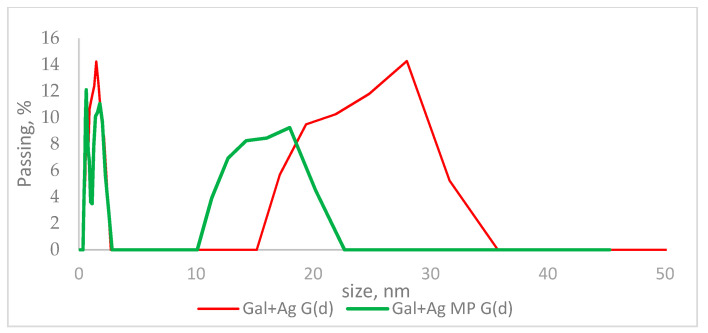
Dynamic light scattering histograms of galactose-assisted Ag NPs and MP-loaded Ag NPs.

The dynamic light scattering histograms show that the median average size of the obtained particles is 27.9 nm for galactose-assisted Ag NPs and 18 nm for MP-loaded Ag NPs (Figure 6).

The zeta potential of galactose-assisted Ag NPs was -21.99 ± 1.53 mV and for drug-loaded Ag NPs it was −10.72 ± 0.74 mV. The negative charge of the zeta potential could refer to carboxylic groups of galactonic acid produced. Carboxylic acids (in this case, the oxidation products of the sugars) are used to provide a negative surface charge density in order to counteract the van der Waals forces responsible for particle coalescence. Self-assembled carboxylic acids ensure dense coating on the metal surfaces and therefore stabilize them [100].

**Figure 7:**
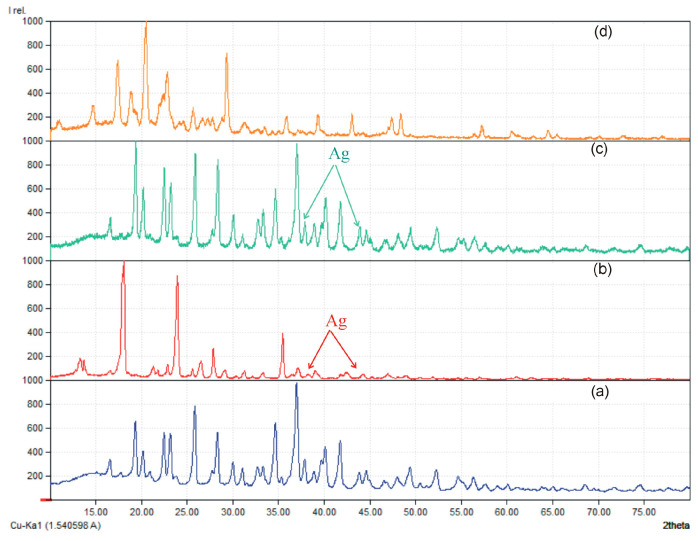
X-ray diffraction patterns of galactose (**a**), Ag NPs (**b**), MP-loaded Ag NPs (**c**), and MP (**d**).

The XRD analysis supports galactose’s role in the synthesis of Ag NPs (Figure 7). Pure galactose ‘s XRD pattern (Figure 7a) and MP’s structure (Figure 7d) revealed multiple distinctive peaks in the 2θ area because of their crystalline structure.

The Ag NPs pattern modified the normal galactose XRD pattern (Figure 7b). At 38.16° and 44.36° of 2θ, the detected signals of Ag NPs are quite weak. The 1 1 1 plane, which can be indexed to the spherical structure of silver [45], corresponds to the strong Bragg reflection at 38.16°.

The silver reflection of MP-loaded Ag NPs (Figure 7c) can be observed at 37.92° and 44.02° of 2θ.

### 3.2. Parametric Drug-Release Optimization

The most popular method of medicine administration is oral. Oral medication is self-administrable, painless, easy to take, and less expensive than intravenous methods. A drug’s oral bioavailability will be hampered if its water solubility is too low. Nanocarriers can be used to resolve such solubility issues [70]. The influence of Ag NPs-to-drug ratio was evaluated, utilizing an in vitro approach with dialysis bags in order to obtain insights into the drug-release trends of MP-loaded Ag NPs [101,102]. Due to a lack of universal, accepted practices, this is a typical technique to learn about innovative drug delivery systems [103]. To determine the in vivo–in vitro correlation of nanoparticle formulations, as well as to direct the development and quality control, drug-release profiles from dialysis-based assays are used [104]. Additionally, they can be utilized to distinguish between different release patterns, such as fast vs. gradual releases [105].

Profiles of MP-molecules only (controls) were compared to those seen in the presence of Ag NPs in order to determine the drug-release properties of the MP-loaded Ag NPs. Different Ag NPs-to-drug ratios (1:1, 1:3, and 1:6) were utilized to examine how the ratio might impact the drug’s release. One drug control per ratio was made using an ethanol solution of MP.

Under normal physiological conditions, the pH value from the stomach to the intestine basically showed an increasing trend, specifically, acidic stomach (pH 1.5 to 3.5), duodenum (pH 6), terminal ileum (pH 7.4), terminal cecum (pH 6), and colon (pH 6.7) [106,107]. To simulate the physiological conditions, the drug-release study was carried out in a neutral medium (PBS, pH 7.4). A cellulose dialysis bag with a molecular weight cut-off of 12,400 Da was used to hold the solution of drug-containing Ag NPs. The pores in this cut-off were large enough to let the medication out of the dialysate while still keeping the Ag NPs inside the membrane. Since the calibration curve for MP was linear, it was possible to conduct the quantification tests.

The length of the intestine, the composition of the gut microbiota, and individual variances in stomach and intestinal fluid and peristalsis may all have a role in the transport time of the gastrointestinal system. There are considerable variations in gastrointestinal emptying periods, not just between healthy participants but also between IBD patients and healthy people, which may raise the uncertainty of when the medicine will reach the colon. Although the small intestine’s average transport time is thought to be 4 h, individual differences often range from 2 to 6 h [107]. Therefore, determining the drug release at the first 6 h was crucial for our investigation.

**Figure 8:**
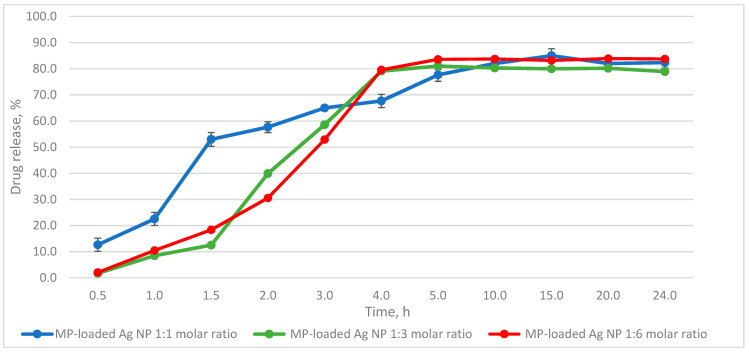
Drug-release concentrations for MP-loaded Ag NPs in molar ratio 1:1, 1:3, and 1:6.

During the 24 h period, the drug release was measured. We found that the absorbance intensity rose with time during the initial five hours of the drug-release experiment, indicating an increasing concentration of the MP in the dialysate (Figure 8).

After the fifth hour, the pattern of drug release changed slightly as the rate of increase in absorbance intensity keep unchanged until the 24th hour. The UV–visible spectra of the released pharmaceuticals in the dialysate, therefore, overlapped in the 24th hour, indicating that the drug release was complete under these circumstances and that the concentration of the released drug in the dialysate was stable. All experiments showed that the dialysate drug concentrations of MP only were a bit higher. That could refer to the protective coat of the galactose and indicates the effectively loaded MP, which slows down the drug release. Nevertheless, MP-loaded Ag NPs indicate a very good drug release between 80 and 85% for all drug ratios at the 6th hour of the experiment. According to the preliminary findings, Ag NPs might be a useful medication delivery mechanism for MP and a potent tool for the treatment of inflammatory bowel disease. For this reason, additional research on long-acting delivery formulations and potential medical uses for MP-loaded Ag NPs will also be required to add to the benefits of this study.

## 4. Conclusions

The rapid synthesis and stabilization of silver nanoparticles using galactose was accomplished utilizing a straightforward, entirely green, one-pot approach. Without the use of microwave irradiation or any other intermediate procedures, the nanoparticles were synthesized in a short amount of time at relatively low temperatures at the water boiling point. The size range for the produced Ag NPs, as determined by various approaches, was 10 to 26 nm for galactose-assisted Ag NPs and 10 to 17 nm for Ag NPs loaded with MP. Galactose was employed as a reducing and capping agent. A thin galactose layer encasing the nanoparticles was visible in the TEM pictures. Additionally established were the Ag NPs’ drug-release capabilities. A valuable perspective for our future investigation is the ex vivo and in vivo determination of the long-acting delivery systems and possible medicinal applications of drug-loaded Ag NPs, namely, in the field of gastrointestinal inflammatory diseases.
